# Job exposure to the public in relation with alcohol, tobacco and cannabis use: Findings from the CONSTANCES cohort study

**DOI:** 10.1371/journal.pone.0196330

**Published:** 2018-05-01

**Authors:** Guillaume Airagnes, Cédric Lemogne, Marcel Goldberg, Nicolas Hoertel, Yves Roquelaure, Frédéric Limosin, Marie Zins

**Affiliations:** 1 Hôpitaux Universitaires Paris Ouest, Department of Psychiatry and Addictology, AP-HP, Paris, France; 2 Faculté de Médecine, Université Paris Descartes, Sorbonne Paris Cité, Paris, France; 3 UMS 011, Population-based Epidemiological Cohorts, Inserm, Villejuif, France; 4 UMR 1168, VIMA, Inserm, Villejuif, France; 5 U 894, Centre Psychiatrie et Neurosciences, Inserm, Paris, France; 6 UMR 1085, Ester, Irest Inserm, Université d’Angers, Angers, France; Universidade Federal de Sao Paulo, BRAZIL

## Abstract

**Objectives:**

To examine the associations between job exposure to the public (e.g., customers, guests, users of a public service, patients) and alcohol, tobacco and cannabis use.

**Methods:**

From the French population-based CONSTANCES cohort, 16,566 men and 17,426 women currently working were included between 2012 and 2016. They reported their exposure to the public (daily versus no daily), and among the daily exposed participants (10,323 men and 13,318 women), the frequency of stressful exposure (often versus rarely). Dependent variables were: chronic alcohol consumption (<1(1), 1-27(1–13), 28-42(14–28), >42(28) drinks per week in men(women)), heavy episodic drinking (never, at most once a month, more than once a month), alcohol use risk with Alcohol Use Disorders Identification Test (mild, dangerous, problematic or dependence), tobacco use (non-smoker, former smoker, 1–9, 10–19, >19 cigarettes per day) and cannabis use (never, not in past year, less than once a month, once a month or more). Logistic regressions provided odds ratios of substance use, stratifying for gender and adjusting for sociodemographic confounders, depression, effort-reward imbalance and perceived health status.

**Results:**

Exposed men had higher risks of alcohol (chronic alcohol consumption, heavy episodic drinking and alcohol use risk), tobacco and cannabis use. Exposed women had higher risks of tobacco and cannabis use. In men, stressful exposure was associated with increased risks of heavy episodic drinking, tobacco and cannabis use. In women, stressful exposure was associated with increased risks of chronic alcohol consumption, alcohol use risk, tobacco and cannabis use. All these findings remained significant in multivariable analyses, taking into account sociodemographic variables, depressive symptoms, perceived health status and effort-reward imbalance.

**Conclusions:**

Interventions to reduce emotional job demand should systematically integrate assessment and prevention measures of addictive behaviors. Vulnerable workers may be offered more specific interventions to reduce the impact of exposure to the public on their substance use.

## Introduction

Addictive behaviors are among the first leading preventable causes of premature death in western countries [1]. The burden of addiction is mainly driven by three substances: alcohol, tobacco and cannabis [2]. Consumption of these substances is involved, with a dose-dependent relationship [3], in many somatic disorders (e.g. cardiovascular disorders, cancers, liver disease) [3, 4], psychiatric disorders (e.g. depression, suicide) [5–8] as well as with social deprivation [9] and detrimental effects on work [10, 11]. For instance, even moderate daily alcohol consumption is associated with sickness absences, whatever their duration and causes of absences [10]. In a recent meta-analysis, quitting smoking was found to reduce absenteeism and lead to substantial cost-savings for employers [11]. However, working conditions may also influence addictive behaviors. For instance, exposure to psychosocial job strain, characterized by high psychological work demand and low decisional latitude to cope with the task, lead to an increased risk of substance use disorders [12], especially concerning alcohol [13, 14], tobacco [15] and cannabis [16]. However, the associations between substance use disorders and emotional job demand has been less explored [17] athough it might constitute a fruitful avenue to refine preventive interventions [12–16, 18].

In the context of job exposure to the public (e.g., customers, guests, users of a public service, patients), emotional job demand involves displaying demands (i.e. expressing positive, negative, and neutral emotions toward public), facing sensitivity demands (i.e. guessing what the public is feeling) and dealing with potential emotional mistreatments (e.g. facing the dissatisfaction of a client) [17]. One may expect an association between these demands and addictive behaviors (i.e. consumption of alcohol, tobacco and cannabis) through several pathways. In one hand, all these situations may induce work-related stress that could lead to adaptive behaviors aiming to help face these interpersonal difficulties, including addictive behaviors [19, 20]. In other words, substances may be used as self-medication to reduce psychological distress induced by these difficulties [19]. In the other hand, substance use disorders may increase the likelihood of interpersonal difficulties [21, 22], either through acute effects or withdrawal symptoms (e.g., irritability) or long-term neuropsychological impairment [23]. In addition, an association between job exposure to the public and addictive behaviors would be consistent with the well-known links between addictive behaviors and social anxiety [24–26]. To our knowledge, no study to date has explored the specific associations between job exposure to the public and alcohol, tobacco and cannabis use in a large population-based sample. Since job strain has been already found to be associated with increased risk of substance use [12–16], it would be particularly interesting to examine whether the associations between substance use and emotional demand is independent of a measure of job strain. Men and women differ regarding their pattern of addictive behaviors. In particular, men have higher levels of consumptions, but women could have a faster progression of the addiction with greater risk for negative health consequences [27]. Men and women could also differ regarding their reasons for substance use. For instance, women might be more prone to experience alcohol-related problems as a consequence of a consumption in social circumstances [28]. Beside addiction, men and women could engage indifferent strategies when coping with emotional job demand. For instance, the association between emotional demand and major depression may be stronger in men, whereas depression is a well-known risk factor for substance use [29]. Another example would be the association between the perceived risk of work accident and emotional demand in women but not in men [17]. Men and women could also be exposed to different types of emotional demand, even for the same job, usually to a greater extent in women [17, 30]. A better understanding of these gender differences would be helpful to build targeted interventions for screening and prevention.

The CONSTANCES cohort include a large randomized sample of the French population, from various occupational status and sociodemographic factors [31]. In particular, sociodemographic characteristics such as age, education level, household income, marital status and occupational status were available, as well as a validated measure of the effort-reward imbalance [32], allowing to control for job strain. In addition, depressive symptoms and perceived health status were also measured. Consequenly we took advantage of the CONSTANCES cohort to examine the cross-sectional associations between job exposure to the public and alcohol, tobacco and cannabis use. All the analyses were examined in men and women, separately, in order to highlight potential gender differences. Two measures of job exposure to the public were considered: daily exposure (versus no daily exposure) and, among participants daily exposed, frequency of stressful exposure. Even without making causal inferences, the identification of these associations, whatever their directions, would result in public health impact. For alcohol, we explored not only the intensity of alcohol intake taking into account both chronic consumption and heavy episodic drinking but also the level of dependency [18]. We hypothesized that such exposure may be associated with increased risk for addictive behaviors for these three substances.

## Material and methods

### Participants

The CONSTANCES cohort includes volunteers aged 18–69 years at baseline in 22 selected health screening centres from the principal regions of France [31]. Participants were selected from French adults being covered by the National Health Insurance Fund according to a random sampling scheme stratified on age, gender, socioeconomic status and region of France. The inclusion visit comprises a set of self-report questionnaires including social and demographic characteristics, health-related behaviors, self-reported health scales, working conditions and occupational exposures.

The present study used the data collected at baseline for the participants included from February 2012 to September 2016, corresponding to 81,997 volunteers. Since our aim was to search for associations between addictive behaviors and job exposure to the public, we selected the sample of 46,652 participants currently working and responders to the assessments of job exposure to the public. Within this subgroup, 33,992 had complete data regarding the selected dependent variables and have been therefore included in the study (S1 Fig). Description of the responders according to missing data and regarding each dependent variable is to be found in S1 Table.

The CONSTANCES cohort has obtained the authorization of the National Data Protection Authority (Commission Nationale de l’Informatique et des Libertés, no. 910486) and was approved by the Institutional Review Board of the National Institute for Medical Research—INSERM (no. 01–011). Written informed consent was received from all of the subjects in the CONSTANCES cohort.

### Job exposure to the public

Two different measures of job exposure to the public were considered:

- *Daily exposure versus no daily exposure*
  A binary question assessed the presence or not of a daily job exposure to the public including a physical or a phone contact with the public every day or almost. What we called public are people other than colleagues with whom the worker has to interact, such as customers, guests, users of a public service or patients.
- *Frequency of stressful exposure among daily exposed workers*
  Daily exposed workers had to answer the following question, exclusively related to their occupational life: « Do you experience stressful situations in your relations with the public? », using a 4-point Likert. More precisely, they had to assess the frequency of stressful exposure by choosing one of the following four responses: 1) Never or almost; 2) Rarely; 3) Often and 4) Always or almost. In the present study, we computed a binary variable by aggregating categories 1) and 2) on the one hand, and categories 3) and 4) on the other hand. Since our outcomes had at least three modalities, we wanted to avoid using too many categories for analysis, which may have resulted in increasing the risk of both Type 1 and 2 errors. Consequently, we introduced the explanatory variable of interest as a binary variable in our analyses (i.e. “never or rarely” versus “often or almost always”).

### Alcohol use

Chronic alcohol consumption and heavy episodic drinking were separately assessed since these different measures of alcohol use have been associated with different environmental factors [22].

Chronic alcohol consumption was computed based on the following question: “How often do you usually drink alcoholic beverages?”. Participants had to choose one of the following four responses: 1) Never; 2) Once a month or less; 3) Two or three times per month and 4) Once a week or more. For participants who declared a chronic consumption once a week or more, weekly alcohol consumption was computed in drinks per week based on a reporting of all the alcoholic beverages consumed the previous week. Weekly alcohol consumption was subsequently categorized in four classes according to the World Health Organization (WHO) levels of risk classification (World Health Organization, 2000). Thus we used the following cut-offs in men (women): <28(14); <43(29); <71(43) and ≥71(43) to define low, medium, high or very high risk categories, respectively. The two last categories (i.e. high and very high) were merged to ensure sufficient subsample size. Then, we computed a unique categorical variable assessing chronic alcohol consumption as follows: 1) Never; 2) Once a month or less; 3) Two or three times per month; 4) low weekly consumption; 5) medium weekly consumption and 6) high or very high weekly consumption. Finally, we aggregated the categories 2) and 3) to provide a meaningful indicator of a non-regular alcohol use (i.e. less than once a week). Since none of our included subjects reported never consuming, our final categorical variable assessing chronic alcohol consumption was thus defined by four categories as follows: 1) Non-regular use; 2) low weekly consumption; 3) medium weekly consumption and 4) high or very high weekly consumption. To also examine whether job exposure to the public would be associated with an increase in weekly alcohol consumption among participants who have a regular consumption (i.e. at least one drink per week), we performed additional analyses in this subsample.

The frequency of heavy episodic drinking was mesured as a categorical variable based on the answer to the following question: « How often do you drink six or more standard alcoholic beverages on the same occasion? ». Participants had to choose among five responses: 1) Never; 2) Less than once per month; 3) Every month; 4) Every week; and 5) Every day or almost. We aggregated categories 2) and 3), as well as categories 4) and 5), in order to compute a categorical variable with 3 modalities as follows: 1) Never, 2) At most once a month and 3) More than once a month. Since frequent heavy drinking was defined by belonging to the last category, we could not examine whether job exposure to the public would be associated with an increase in the frequency of heavy episodic drinking among a subsample of frequent heavy episodic drinkers.

Finally, alcohol use risk categories were defined thanks to the French version of the Alcohol Use Disorders Identification Test (AUDIT) [33]. The AUDIT was developed in 1989 by the World Health Organization (WHO) and has been updated in 1992 to match the DSM-IV criteria for alcohol abuse and dependence. It consists of a 10-item self-administered questionnaire built as a transcultural screening tool about recent alcohol use, alcohol dependence symptoms and alcohol-related problems. The AUDIT score ranges from 0 to 40. In the present study, the AUDIT was used as a categorical variable with four modalities, based on recommended AUDIT risk levels, and in order to provide a meaningful indicator of alcohol use disorder severity, as follows: 1) Mild (0–7); 2) Dangerous (8–15); 3) Problematic (16–19) and 4) Dependence (20–40) [34]. The two last categories (i.e. problematic and dependence) were merged to ensure sufficient subsample size.

### Tobacco use

Smoking status (i.e. non-smoker, former smoker or current smoker) was self-reported. Among current smokers, daily tobacco consumption was computed in cigarettes per day from the cumulative number of tobacco consumption per day, whatever the product (e.g. common cigarette, cigar and pipe). From these two variables, we computed a categorical variable with five modalities to define 1) Non-smokers; 2) Former smokers; 3) Current light smokers (1 to 9 cigarettes per day); 4) Current moderate smokers (10 to 19 cigarettes per day) and 3) Current heavy smokers (>19 cigarettes per day) [35]. To also examine whether job exposure to the public would be associated with an increase in tobacco consumption among smokers, we performed additional analyses in the subsample of current smokers.

### Cannabis use

From three questions asked to characterize the frequency of cannabis consumption, we computed a categorical variable expressing the frequency of lifetime cannabis consumption as follows: 1) Never used; 2) No consumption during the previous 12 months; 3) Less than once a month and 4) Once a month or more. Since regular cannabis consumption was defined by belonging to the last category, we could not examine whether job exposure to the public would be associated with an increase in cannabis consumption among a subsample of regular users.

### Covariables

From the baseline questionnaires, we used the following sociodemographic variables:—age; gender; occupational status indicated as follows: farmer, blue-collar worker and craftsman; clerk (e.g. clerical or commercial employee, childcare worker, service agent); intermediate worker (e.g. school teacher, nurse, technician, foreman, master’s agent); executive (e.g. engineer, doctor);—marital status (i.e. single; marital life; separated or divorced; widowed); household income (i.e. <2100; 2100–2800; 2800–4200; >4200 euros per month);—education level based on the International Standard Classification of Education (ISCED) [36]. Education level has been coded according to the nine levels of the International Standard Classification of Education (ISCED) [36]. In the CONSTANCES cohort, a categorical variable corresponding to the highest obtained degree can be directly used to compute an education level categorical variable in five modalities based on the 2011 ISCED classification as follows: levels 0 and 1 (early childhood education and primary education); level 2 (lower secondary education); levels 3 and 4 (upper secondary education and post-secondary non-tertiary education); levels 5 and 6 (short-cycle tertiary education and Bachelor’s or equivalent level) and levels 7 and 8 (Master’s or equivalent level and Doctoral or equivalent level). Except for age, these variables were collected as categorical ones.

Since depression has been found to be associated with both substance consumptions [5–8] and occupational stress [37], we also collected depressive symptoms as a continuous variable with the Center of Epidemiologic Studies Depression scale (CESD), which is a 20-item self-administered questionnaire designed for use in community studies. The CESD is known to have a high internal consistency [38]. The CESD asks participants how often they have experienced specific symptoms during the previous week (e.g. «I felt depressed», «I felt everything I did was an effort», «My sleep was restless»). Responses range from 0 («hardly ever») to 3 («most of the time»).

Perceived health status is associated with substance use disorder as well as with social anxiety [39]. In the present study, perceived health status has been assessed by fulfilling the following question: « How do you judge your general health compared to a person of your entourage of the same age? ». The score ranges from 1 to 8, with a score of 1 indicated a very good general health and a score of 8 a very poor one.

As job strain has been associated with substance use and may increase the sensitivity to stressful situations in the workplace [12], we used the effort-reward imbalance as a continuous variable to provide a proxy of sustained stress reactions at work. First, participants answered three questions regarding their efforts at work (e.g. demanding job, heavy workload) and seven questions regarding their rewards at work (e.g. financial gratification, respect from superiors) by completing a 4-point likert scale for each sentence according to their level of agreement with the proposal (from “strongly disagree” to “totally agree”). Scores at each question goes from 1 to 4 points. For three questions regarding rewards at work, scores had to be inversed. Secondly, two different total scores, one regarding efforts and another regard rewards, were computed by summing the scores at each question. Thirdly, the effort-reward imbalance was computed, using the following formula: (7/3)*(effort total score/reward total score). The effort-reward ratio ranges from 0.25 to 4. Finally, for the participants daily exposed to the public, we performed exploratory analyses to examine whether the association between substance use and stressful exposure to the public might be more prevalent in some types of job. The CONSTANCES cohort includes information about the profession for some participants in the labor force using an automatic coding from the National Institute of Statistics and Economic Studies [40]. These data were available for 7,991 men and 11,411 among the daily exposed workers. To prevent Type 2 error because of insufficient statistical power, we took into account only the categories of jobs including a sufficient number of participants. This was not the case for clergy (3 men and no woman), information, arts and entertainment professions (111 men and 158 women), farmers (one man and one woman) and company managers (6 men and no woman). The remaining seven categories of jobs, including a total of 7,703 men and 10,916 women were: administrative staff, education professions, healthcare professionals, supervisors, workers and technicians who are not in the tertiary sector, engineers, personal services, and commercial and independent professions (S2 Table).

### Statistical analysis

Our dependent variables (chronic alcohol consumption, heavy episodic drinking, alchol use risk, tobaco consumption and cannabis consumption) were either available as categorical ones or divided into clinically relevant categories to provide meaningful risk estimates (see above). These dependent variables were used in separate multinomial logistic regressions. Results are presented as estimated odds ratios (OR) with their 95% confidence intervals (CI).

First, for each dependent variable, we introduced daily exposure to the public as a fixed factor into an age-adjusted model and then, into a model adjusted for occupational status and marital status as fixed factors and for age at baseline, household income and education level as continuous covariables. We indeed assumed that these two last variables were ordinal representation of underlying sets of continuous units (i.e. years of education and amount of money in euros per month) [41]. Second, the analyses described above were repeated in the subsample of daily exposed participants, using frequency of job exposure to the public as the explanatory variable of interest. Third, we performed exploratory age-adjusted analyses while stratifying this subsample for gender and type of jobs.

As sensitivity analysis, we performed additional adjustement for either depressive symptoms or perceived health status or effort-reward imbalance, as continous variables. In order to examine the risk of listwise deletion by including subjects with complete data for the outcomes, we ran the analyses again including all the responders after dealing with missing data either by multiple imputations or by multiple imputation then deletion [42]. As specificity analysis, we repeated the main analyses replacing the frequency of stressful exposure the public with the intensity of physical effort at work, as a control condition. Tertiles of perceived physical job demand were computed from the following 8-item likert scale: « How do you assess the intensity of the physical efforts of your work during a typical day of work? », a variable which is a good proxy of a physically demanding job [43].

Included subjects had complete data regarding the dependent variables and the explanatory variable of interest. We had missing data for the other variables, except for gender, occupational status and age at baseline. Assuming a missing at random mechanism, imputation was preferred to complete-case analysis in order to limit the risk of selection bias (S3 Table). Stochastic regression imputations were used since this approach accounts for some additional variance in the imputed estimates compared to simple regression [44].

All the analyses were stratified by gender. Because of the exploratory component of the study, statistical significance was determined using a conservative two-sided alpha a priori set at 0.05 and analyses were performed with IBM Statistics for Windows, Version 22.0, Released 2013 (Armonk, NY: IBM Corp).

## Results

### Participants’ characteristics

The characteristics of the included participants (16,566 men and 17,426 women) stratified by gender are displayed in Table 1. Among them, 12,124 men and 9,353 women were regular alcohol users, and 3,179 men and 3,225 women were current smokers. Among these included participants, 10,323 men and 13,318 women had a daily job exposure to the public. Among these daily exposed participants, 2,759 men and 4,152 women experienced frequent stressful exposure, 7,627 men and 7,071 women were regular alcohol users, and 2,121 men and 2,580 women were current smokers.

**Table 1:** Characteristics of the 33,992 included participants.

GENDER	All included participants				Participants with a daily job exposure to the public			
	MEN		WOMEN		MEN		WOMEN	
N (%)	16,566(48.7%)		17,426(51.3%)		10,323(43.7%)		13,318(56.3%)	
**CONTINUOUS VARIABLES**	**Mean**	**SD**	**Mean**	**SD**	**Mean**	**SD**	**Mean**	**SD**
Age (years)	44.3	10.3	43.7	10.4	44.0	10.3	43.5	10.4
Depression score (CESD)	9.0	7.3	11.4	8.9	9.1	7.4	11.5	8.9
Perceived health status^a^	2.7	1.3	2.7	1.3	2.7	1.3	2.7	1.3
Effort-reward imbalance^b^	1.0	0.4	1.1	0.4	1.1	0.4	1.1	0.4
**CATEGORICAL VARIABLES**	**N**	**%**	**N**	**%**	**N**	**%**	**N**	**%**
**Occupational status**								
Farmer, blue-collar worker and craftsman	2512	15.2	558	3.2	1314	12.7	288	2.2
Clerk	2382	14.4	5801	33.3	1880	18.2	4790	36.0
Intermediate worker	4358	26.3	5948	34.1	3186	30.9	5150	38.7
Executive	7314	44.2	5119	29.4	3943	38.2	3090	23.2
**Marital status**								
Single	2341	14.1	2707	15.5	1417	13.7	1999	15.0
Marital life	12768	77.1	12343	70.8	7959	77.1	9473	71.1
Separated or divorced	1380	8.3	2112	12.1	894	8.7	1637	12.3
Widower	77	0.5	264	1.5	53	0.5	209	1.6
**Household income**(euros per month)								
Less than 2100	2252	13.6	3191	18.3	1520	14.7	2577	19.3
Between 2100 and 2800	2407	14.5	2854	16.4	1572	15.2	2239	16.8
Between 2800 and 4200	5708	34.5	5392	34.0	3725	36.1	4708	35.4
More than 4200	6199	37.4	5449	31.3	3506	34.0	3794	28.5
**Education ISCED classification**								
Levels 0 and 1	302	1.8	217	1.2	174	1.7	135	1.0
Level 2	551	3.3	583	3.3	358	3.5	443	3.3
Levels 3 and 4	5232	31.6	4559	26.2	3417	33.1	3604	27.1
Levels 5 and 6	5372	32.4	7668	44.0	3773	36.5	6330	47.5
Levels 7 and 8	5109	30.8	4399	25.2	2601	25.2	2806	21.1
**Daily job exposure to the publicto the public in the workplace**								
No	6243	37.7	4108	23.6	0	0	0	0
Yes	10323	62.3	13318	76.4	10323	100	13318	100
**Frequency of stressful exposure**								
Never or rarely	7564	73.3	9166	68.8	7564	73.3	9166	68.8
Often or almost always	2759	26.7	4152	31.2	2759	26.7	4152	31.2
**Chronic alcohol consumption**^c^								
Non regular user	4442	26.8	8073	46.3	2696	26.1	6247	46.9
Low	11165	67.4	8144	46.7	6952	67.3	6172	46.3
Medium	666	4.0	1025	5.9	473	4.6	764	5.7
High or very high	293	1.8	184	1.1	202	2.0	135	1.0
**Heavy episodic drinking**^d^								
Never	5823	35.2	11459	65.8	3473	33.6	8819	66.2
At most once a month	9220	55.7	5486	31.5	5839	56.6	4150	31.2
More than once a month	1523	9.2	481	2.8	1011	9.8	349	2.6
**Alcohol use risk**^e^								
Mild	13070	78.9	16157	92.7	8013	77.6	12369	92.9
Dangerous	3023	18.2	1136	6.5	1996	19.3	851	6.4
Problematic or dependence	473	2.9	133	0.8	314	3.0	98	0.7
**Tobacco use**^f^								
Non-smoker	7262	43.8	8378	48.1	4312	41.8	6318	47.4
Former smoker	6125	37.0	5823	33.4	3890	37.7	4420	33.2
Current light smoker	1250	7.5	1616	9.3	819	7.9	1285	9.6
Current moderate smoker	1347	8.1	1328	7.6	906	8.8	1064	8.0
Current heavy smoker	582	3.5	281	1.6	396	3.8	231	1.7
**Cannabis use**								
Never used	7473	45.1	9856	56.6	4508	43.7	7462	56.0
No consumption during the previous 12 months	7233	43.7	6523	37.4	4557	44.1	5052	37.9
Less than once a month	817	4.9	580	3.3	550	5.3	459	3.4
Once a month or more	1043	6.3	467	2.7	708	6.9	345	2.6

SD: Standard Deviation; CESD: Center for Epidemiologic Studies Depression Scale; ISCED: International Standard Classification of Education;

^a^From a 8-pointLikert scale with a score of 1 indicated a very good general health and a score of 8 a very poor one;

^b^Computed from 7 items regarding rewards and from 3 items regarding efforts as follows: ERI = (7/3)*(effort total score/reward total score), and with all the items assessed on a 4-pointlikert scale;

^c^A non-regular consumption is defined as a consumption of less than one standard drink per week. Regarding regular consumers, the following cut-offs in men(women):<28drinks per week(14); <43(29); <71(43) and ≥71(43) define low, medium, high or very high risk alcohol consumption categories, respectively;

^d^Defined as at least six standard alcoholic beverages on the same occasion;

^e^Categories are defined from Alcohol Use Disorders Identification scores as follows: Mild (0–7), Dangerous (8–15), Problematic (16–19) and Dependence (20–40);

^f^Categories of current smokers are defined as follows: Light (1 to 9 cigarettes per day), Moderate (10 to 19) and Heavy (>19) consumers.

### Relations between daily job exposure to the public and alcohol, tobacco and cannabis use

In men, daily exposure (versus no daily exposure) was positively associated with all the outcomes (i.e. chronic alcohol consumption, heavy episodic drinking, alcohol use risk, tobacco consumption and cannabis consumption). These associations concerned all the categories of consumers and remained significant after adjustments for all sociodemographic variables. Except for cannabis consumption, we found gradually increased risks according to the intensity of consumption (Table 2).

**Table 2:** Associations between alcohol, tobacco and cannabis use and daily job exposure to the public, taking no daily exposure as reference category, in 16,566 men and 17,426 women.

MEN	Age-adjusted				Adjusted for all sociodemographic variables			
	OR	95%CI		p value	OR	95%CI		p value
**Chronic alcohol consumption**^a^								
Low	**1.08**	**1.00**	**1.16**	**0.039**	**1.15**	**1.06**	**1.23**	**<0.001**
Moderate	**1.61**	**1.35**	**1.92**	**<0.001**	**1.71**	**1.43**	**2.05**	**<0.001**
High or very high	**1.46**	**1.13**	**1.88**	**0.004**	**1.55**	**1.19**	**2.01**	**0.001**
**Heavy episodic drinking**^b^								
At most once a month	**1.14**	**1.07**	**1.22**	**<0.001**	**1.16**	**1.08**	**1.25**	**<0.001**
More than once a month	**1.30**	**1.15**	**1.46**	**<0.001**	**1.33**	**1.18**	**1.51**	**<0.001**
**Alcohol use risk**^c^								
Dangerous	**1.20**	**1.10**	**1.31**	**<0.001**	**1.22**	**1.12**	**1.33**	**<0.001**
Problematic or Dependence	**1.22**	**1.00**	**1.48**	**0.049**	**1.24**	**1.02**	**1.52**	**0.035**
**Smoking status**^d^								
Former smoker	**1.23**	**1.15**	**1.32**	**<0.001**	**1.17**	**1.09**	**1.26**	**<0.001**
Light smoker	**1.26**	**1.11**	**1.43**	**<0.001**	**1.19**	**1.05**	**1.36**	**0.008**
Moderate smoker	**1.38**	**1.22**	**1.57**	**<0.001**	**1.26**	**1.11**	**1.44**	**<0.001**
Heavy smoker	**1.48**	**1.24**	**1.77**	**<0.001**	**1.47**	**1.22**	**1.78**	**<0.001**
**Cannabis consumption**^e^								
Consumption more than 12 months ago	**1.10**	**1.03**	**1.18**	**0.006**	**1.08**	**1.01**	**1.16**	**0.031**
Less than once a month	**1.29**	**1.10**	**1.51**	**0.002**	**1.38**	**1.17**	**1.63**	**<0.001**
Once a month or more	**1.32**	**1.14**	**1.52**	**<0.001**	**1.25**	**1.08**	**1.45**	**0.003**
**WOMEN**								
**Chronic alcohol consumption**^a^								
Low	**0.92**	**0.86**	**0.99**	**0.029**	1.04	0.96	1.12	0.310
Moderate	**0.86**	**0.74**	**0.99**	**0.043**	1.03	0.88	1.20	0.734
High or very high	0.80	0.58	1.12	0.187	0.96	0.68	1.36	0.810
**Heavy episodic drinking**^b^								
At most once a month	**0.89**	**0.82**	**0.96**	**0.003**	0.96	0.89	1.05	0.363
More than once a month	**0.77**	**0.62**	**0.94**	**0.011**	0.84	0.67	1.04	0.104
**Alcohol use risk**^c^								
Dangerous	0.88	0.77	1.01	0.075	1.01	0.87	1.17	0.919
Problematic or Dependence	0.84	0.57	1.23	0.369	1.01	0.67	1.52	0.964
**Smoking status**^d^								
Former smoker	1.05	0.97	1.13	0.251	1.03	0.95	1.12	0.442
Light smoker	**1.25**	**1.09**	**1.42**	**0.001**	**1.20**	**1.04**	**1.37**	**0.010**
Moderate smoker	**1.30**	**1.13**	**1.50**	**<0.001**	**1.25**	**1.07**	**1.45**	**0.005**
Heavy smoker	**1.53**	**1.12**	**2.09**	**0.007**	**1.63**	**1.18**	**2.25**	**0.003**
**Cannabis consumption**^e^								
Consumption more than 12 months ago	1.07	0.99	1.16	0.063	**1.10**	**1.02**	**1.19**	**0.019**
Less than once a month	1.12	0.91	1.38	0.284	**1.31**	**1.05**	**1.63**	**0.017**
Once a month or more	0.83	0.67	1.03	0.092	0.87	0.69	1.09	0.229

OR: Odd ratio; 95%CI: Confidence interval at 95%; %;

^a^ Reference category is non-regular consumption, i.e. consumption of less than one standard drink per week. Regarding regular consumers, the following cut-offs in men(women):<28drinks per week(14); <43(29); <71(43) and ≥71(43) define low, medium, high or very high risk alcohol consumption categories, respectively;

^b^ Defined as at least six standard alcoholic beverages on the same occasion and taking the "never" category as reference;

^c^ Categories are defined from Alcohol Use Disorders Identification scores as follows: Mild (0–7), Dangerous (8–15), Problematic (16–19) and Dependence (20–40), with Mild category as reference;

^d^ Categories of current smokers are defined as follows: Light (1 to 9 cigarettes per day), Moderate (10 to 19) and Heavy (>19) consumers, with non-smokers as reference category;

^e^ Reference category is never use. Age at baseline was used as continuous covariable. Other sociodemographic variables include occupational status and marital status as fixed factors and household income and education level as continuous covariables. Significant associations are presented in bold (i.e. p<0.05).

In women, daily exposure (versus no daily exposure) was positively associated with current smoking compared to non-smoking. This association remained significant after adjustment for all sociodemographic variables, with gradually increased risks according to the intensity of consumption. Cannabis consumption was positively associated with daily exposure only after adjustment for all the sociodemographic variables (Table 2).

When considering the subsample of participants with regular alcohol consumption, daily exposure was positively associated with the risk of being a moderate or a high or very high consumer compared to a low one, in men but not in women (Table 3). When considering the subsample of current smokers, daily exposure was positively associated with the risk of being a heavy smoker compared to a light one, in men but not in women (Table 3). These associations were significant after adjustment for all sociodemographic variables.

**Table 3:** Associations between job exposure to the public and alcohol and tobacco use, among regular alcohol users and current smokers, respectively.

SAMPLE	All participants^a^								Daily exposed participants^b^							
INDEPENDENT VARIABLE OF INTEREST	Daily exposure (compared to no daily exposure)								Frequent stressful exposure (compared to rare stressful exposure)							
	Age-adjusted				Adjusted for all sociodemographic variables				Age-adjusted				Adjusted for all sociodemographic variables			
MEN	OR	95%CI		p value	OR	95%CI		p value	OR	95%CI		p value	OR	95%CI		p value
**Alcohol consumption**^c^																
Moderate	**1.49**	**1.26**	**1.77**	**<0.001**	**1.50**	**1.25**	**1.78**	**<0.001**	0.99	0.81	1.12	0.976	1.02	0.82	1.26	0.885
High or very high	**1.35**	**1.05**	**1.74**	**0.019**	**1.35**	**1.04**	**1.74**	**0.024**	1.08	0.79	1.47	0.635	1.12	0.82	1.54	0.473
**Tobacco consumption**^d^																
Moderate smoker	1.12	0.95	1.32	0.175	1.09	0.92	1.30	0.325	1.14	0.92	1.42	0.241	1.15	0.92	1.44	0.210
Heavy smoker	**1.28**	**1.03**	**1.58**	**0.027**	**1.36**	**1.09**	**1.72**	**0.008**	1.20	0.91	1.59	0.202	1.29	0.97	1.72	0.086
**WOMEN**																
**Alcohol consumption**^c^																
Moderate	0.9	0.80	1.08	0.366	0.98	0.84	1.15	0.841	**1.20**	**1.03**	**1.41**	**0.023**	**1.21**	**1.03**	**1.42**	**0.020**
High or very high	0.88	0.63	1.22	0.435	0.92	0.65	1.30	0.642	**1.62**	**1.14**	**2.29**	**0.007**	**1.64**	**1.16**	**2.32**	**0.006**
**Tobacco consumption**^d^																
Moderate smoker	1.05	0.87	1.26	0.614	1.04	0.86	1.26	0.663	1.12	0.95	1.33	0.190	1.16	0.98	1.39	0.092
Heavy smoker	1.25	0.90	1.74	0.190	1.34	0.95	1.89	0.100	**1.54**	**1.15**	**2.05**	**0.003**	**1.58**	**1.18**	**2.12**	**0.002**

OR: Odd ratio; 95%CI: Confidence interval at 95%; %;

^a^Among 12,124 men and 9,253 women for alcohol regular users (i.e. at least one standard drink per week) or among 3,179 men and 3,225 women for current smokers;

^b^ Among 7,627 men and 7,071 women for alcohol regular users (i.e. at least one standard drink per week) or among 2,121 men and 2,580 women for current smokers;

^c^ Reference category is low consumption, the following cut-offs in men(women):<28drinks per week(14); <43(29); <71(43) and ≥71(43) define low, medium, high or very high risk alcohol consumption categories, respectively;

^d^Reference category is light consumption, categories of current smokers are defined as follows: Light (1 to 9 cigarettes per day), Moderate (10 to 19) and Heavy (>19) consumers, with non-smokers as reference category. Age at baseline was used as continuous covariable. Other sociodemographic variables include occupational status and marital status as fixed factors and household income and education level as continuous covariables. Significant associations are presented in bold (i.e. p<0.05).

### Relations between stressful job exposure to the public and alcohol, tobacco and cannabis use

In men, a stressful exposure was positively associated with increased risks of heavy episodic drinking, being a former smoker and cannabis consumption (Table 4). These associations remained significant after adjustments for all sociodemographic variables. Stressful exposure was associated with alcohol use risk only after adjustment for all sociodemographic variables.

**Table 4:** Associations between alcohol, tobacco and cannabis use and frequency of stressful job exposure to the public among 10,323 men and 13,318 women, all daily exposed, and considering a frequent stressful exposure compared to a rare one.

MEN	Age-adjusted				Adjusted for all sociodemographic variables			
	OR	95%CI		p value	OR	95%CI		p value
**Chronic alcohol consumption**^a^								
Low	0.99	0.90	1.10	0.853	0.99	0.90	1.10	0.966
Moderate	0.99	0.79	1.23	0.914	1.02	0.81	1.27	0.895
High or very high	1.07	0.78	1.47	0.681	1.12	0.82	1.55	0.474
**Heavy episodic drinking**^b^								
At most once a month	1.02	0.93	1.13	0.632	1.03	0.93	1.13	0.623
More than once a month	**1.26**	**1.07**	**1.47**	**0.004**	**1.29**	**1.10**	**1.51**	**0.002**
**Alcohol use risk**^c^								
Dangerous	1.06	0.95	1.19	0.305	1.08	0.96	1.21	0.189
Problematic or Dependence	1.23	0.96	1.58	0.098	1.28	0.99	1.65	0.052
**Smoking status**^d^								
Former smoker	**1.17**	**1.06**	**1.30**	**0.002**	**1.17**	**1.06**	**1.29**	**0.002**
Light smoker	0.92	0.77	1.10	0.363	0.91	0.76	1.09	0.300
Moderate smoker	1.04	0.88	1.22	0.659	1.03	0.88	1.22	0.693
Heavy smoker	1.05	0.83	1.32	0.701	1.10	0.87	1.40	0.432
**Cannabis consumption**^e^								
Consumption more than 12 months ago	**1.17**	**1.07**	**1.29**	**0.001**	**1.17**	**1.06**	**1.29**	**0.001**
Less than once a month	1.11	0.91	1.37	0.304	1.15	0.93	1.42	0.188
Once a month or more	1.06	0.88	1.28	0.564	1.05	0.87	1.27	0.628
**WOMEN**								
**Chronic alcohol consumption**^a^								
Low	0.97	0.90	1.04	0.377	0.97	0.90	1.05	0.446
Moderate	1.16	0.99	1.36	0.062	1.17	1.00	1.38	0.050
High or very high	**1.57**	**1.11**	**2.21**	**0.011**	**1.59**	**1.12**	**2.25**	**0.009**
**Heavy episodic drinking**^b^								
At most once a month	1.06	0.97	1.15	0.189	1.06	0.97	1.15	0.182
More than once a month	1.22	0.97	1.52	0.087	1.25	0.99	1.57	0.055
**Alcohol use risk**^c^								
Dangerous	**1.32**	**1.14**	**1.53**	**<0.001**	**1.34**	**1.16**	**1.56**	**<0.001**
Problematic or Dependence	**2.20**	**1.48**	**3.27**	**<0.001**	**2.30**	**1.54**	**3.44**	**<0.001**
**Smoking status**^d^								
Former smoker	1.09	0.99	1.18	0.057	1.09	1.00	1.18	0.051
Light smoker	1.13	0.99	1.29	0.063	1.13	0.99	1.29	0.061
Moderate smoker	**1.27**	**1.10**	**1.45**	**0.001**	**1.30**	**1.13**	**1.49**	**<0.001**
Heavy smoker	**1.71**	**1.31**	**2.23**	**<0.001**	**1.75**	**1.34**	**2.30**	**<0.001**
**Cannabis consumption**^e^								
Consumption more than 12 months ago	**1.23**	**1.14**	**1.33**	**<0.001**	**1.23**	**1.14**	**1.33**	**<0.001**
Less than once a month	1.02	0.83	1.27	0.830	1.03	0.83	1.28	0.782
Once a month or more	**1.39**	**1.11**	**1.75**	**0.005**	**1.38**	**1.10**	**1.75**	**0.006**

OR: Odd ratio; 95%CI: Confidence interval at 95%; %;

^a^ Reference category is non-regular consumption, i.e. consumption of less than one standard drink per week. Regarding regular consumers, the following cut-offs in men(women):<28drinks per week(14); <43(29); <71(43) and ≥71(43) define low, medium, high or very high risk alcohol consumption categories, respectively;

^b^ Defined as at least six standard alcoholic beverages on the same occasion and taking the "never" category as reference;

^c^ Categories are defined from Alcohol Use Disorders Identification scores as follows: Mild (0–7), Dangerous (8–15), Problematic (16–19) and Dependence (20–40), with Mild category as reference;

^d^ Categories of current smokers are defined as follows: Light (1 to 9 cigarettes per day), Moderate (10 to 19) and Heavy (>19) consumers, with non-smokers as reference category;

^e^ Reference category is never use. Age at baseline was used as continuous covariable. Other sociodemographic variables include occupational status and marital status as fixed factors and household income and education level as continuous covariables. Significant associations are presented in bold (i.e. p<0.05).

In women, stressful exposure was positively associated with increased risks of chronic alcohol consumption, alcohol use risk, tobacco and cannabis consumption (Table 4). These associations remained significant after adjustments for all sociodemographic data. We found gradually increased risks according to the category of alcohol use risk and the intensity of tobacco consumption.

When considering the subsample of participants with regular alcohol consumption, stressful exposure was positively associated with chronic alcohol consumption in women but not in men, and with gradually increased risks (Table 3). When considering the subsample of current smokers, stressful exposure was positively associated with the risk of being a heavy smoker compared to a light one in women but not in men (Table 3). These associations remained significant after adjustement for all sociodemographic variables.

When examining which types of jobs might be mainly concerned by the increased risk of substance use according to stressful exposure, we found education in men, and education and healthcare in women (S2 Table).

### Sensitivity analysis

Additional adjustements for depressive symptoms, perceived health status and effort-reward imbalance did not change the results. After multiple imputations as well as after multiple imputation then deletion among all the responders, we found similar significant associations as those described within the included subjects (data not shown).

### Specificity analysis

To challenge the specificity of our findings, we substituted our independent variable of interest (i.e. frequency of stressful exposure to the public in the workplace) by tertiles of perceived physical job demand. In men, being in the third tertile compared to the first was associated with being a moderate alcohol consumer (OR = 1.29(1.01–1.63), p = 0.038), being in the problematic or dependence category of alcohol use risk (OR = 1.32(1.01–1.73), p = 0.041) and being a heavy tobacco consumer (OR = 1.72(1.27–2.32), p<0.001). In men, being in the second tertile compared to the first was associated with having heavy episodic drinking at most once a month (OR = 1.10(1.02–1.20), p = 0.019). In women, being in the third tertile compared to the first was associated with being a low alcohol consumer (OR = 0.85(0.79–0.92), p<0;001), being a heavy smoker (OR = 1.42(1.03–1.96), p = 0.031), having cannabis consumption more than 12 months ago (OR = 1.12(1.03–1.21), p = 0.009) and less than once a month (OR = 0.75(0.59–0.95), p = 0.015). Being in the second tertile compared to the first was associatd with having cannabis consumption less than once a month (OR = 0.72(0.59–0.89), p = 0.002). In women, associations were either positive or negative. There were no other significant associations.

## Discussion

### Summary of the results

The main objective of the study was to examine the associations between job exposure to the public and alcohol, tobacco and cannabis use, among a large population-based sample in men and women, separately. In addition, we used two measures of job exposure to the public: daily exposure (versus no daily exposure) and frequency of stressful exposure. Exposed men had higher risks of alcohol (chronic alcohol consumption, heavy episodic drinking and alcohol use risk), tobacco and cannabis use. Moreover, in men, regular alcohol users had a higher risk of increasing their alcohol consumption, as well as current smokers regarding their tobacco consumption. Exposed women had higher risks of tobacco and cannabis use. In men, stressful exposure was associated with increased risks of heavy episodic drinking, tobacco and cannabis use. In women, stressful exposure was associated with increased risks of chronic alcohol consumption, alcohol use risk, tobacco and cannabis use. Moreover, in women, regular alcohol users had a higher risk of increasing their alcohol consumption, as well as current smokers regarding their tobacco consumption. All these findings were specific to exposure to the public and not to physical job demand. They remained significant in multivariable analyses, taking into account sociodemographic variables, depressive symptoms, perceived health status and effort-reward imbalance.

### Strength and limitations

To our knowledge, this is the first study to explore the specific associations between job exposure to the public and several addictive behaviors, including cannabis use, in a large population-based sample of randomly recruited men and women, taking into account several confounding factors (sociodemographic factors, depressive symptoms, perceived health status and job strain). Two different aspects of exposure to the public were examined: exposure per se and stressful exposure. In addition, three different aspects of alcohol use, which relate to different risk factors and hazards, were examined, namely weekly consumption, heavy episodic drinking frequency, and the risk of alcohol use disorder, as assessed with a validated tool.

Our study has several limitations. Firstly, the cross-sectional design does not allow determining the direction of the association. Secondly, even if the CONSTANCES cohort randomly recruited its participants, this population is not representative of the general population due to selection effects associated with voluntary participation. Thus, our findings may not apply to the same extent to other settings. Thirdly, the listwise deletion of individuals who had missing data for dependent variables led to a decrease in statistical power and potentially a selection of subjects less likely to display severe addictive behaviors. Therefore our results might have underestimated the weight of stressful exposure to the public in the associations with alcohol, tobacco and cannabis use. Because of multiple tests performed, one could be concerned with an inflated alpha risk. However, because of the well-known co-occurrence of addictive behaviors, the tests were not formally independent. Furthermore, the results were generally consistent over the different dependent variables, were in line with a priori hypotheses and remained similar after dealing with missing data in different ways. Finally, it is noteworthy that specificity analysis found non-significant results when taking another occupational risk as explanatory variable. Fourthly, daily exposure was assessed with a binary variable, which precluded examining the role of less frequent exposures. Fifhtly, stressful exposure was assessed by a unique question whose wording might not be understood in the same way by all the participants. In particular, men and women may differ about what they consider as « stressful situations », which could explain, at least partially, the observed gender differences. More generally, this measure did not allow us to examine the role of individual sensitivity to this stressful exposure, which may differently relate to different substances and use patterns.

### Explanatory hypotheses

Although the cross-sectional design of our study does not allow drawing causal interpretations, causal relationships may nonethess explain, at least partially, our results. In one hand, job exposure to the public may increase the risk of substance use disorders [24, 26]. One could hypothesize that substances may be used as self-medication in the hope of decreasing stress and anxiety induced by this emotional job demand [19]. For instance, alcohol may have acute anxiolytic effect through GABA-mediated pathways. Interestingly, a daily exposure to the public, regardless of its stressful nature, was associated with increased risks of substance use. This finding should be interpreted in the light of the complex relationships between emotional demand and work stress [45]. An emotional demand could indeed arise even without experiencing a stressful situation per se (e.g. having to show smiles and cheerfulness whatever one’s inner emotional state). In addition, since the attitude of the public is often unpredictable, another example would be anticipatory anxiety and efforts to avoid strained relations which might constitute substantial emotional demands. Finally, differences in substance use between exposed and non-exposed workers might be explained, at least partially, by other sociodemographic or job-related factors. Furthermore, some non-exposed workers could have been withdrawn of this exposure due to interpersonal difficulties, including those potentially resulted from addictive behaviors. This potential healthy worker effect might lead to underestimate the strength of the associations. In the other hand, substance use disorders may increase the likelihood of interpersonal difficulties. Fisrt, acute effects or withdrawal symptoms may increases impulsivity and irritability while decreasing the abilty to perform work-related tasks in relation with the public. Second, chronic substance use may induce long-term neuropsychological impairment [23]. These cognitive deficits that may be linked to subsequent social difficulties may differ following the pattern of alcohol intake, i.e. chonic use versus heavy episodic intake [46]. Obviously, both short-term and long-term effects may increase the likelihood of dissatisfaction of the public, triggering interpersonal difficulties.

Our results are in accordance with our a priori hypotheses based on previous findings regarding the associations between work stress and increased addictive behaviors [12, 13, 15, 16]. They are also in line with the literature on the links between interpersonal difficulties and addictive behaviors such as in social anxiety [24–26]. In addition, our results extend the available literature since we examined in the same sample the associations between the three most harmful substances at a population level and a widespread occupational risk involving work-related interpersonal difficulties. We have also examined the risks for increased consumptions among subgroups of regular alcohol users and current smokers. In addition, we were able to examine the specific role of exposure to the public controlling for job strain, using a validated tool based on the effort-reward model. Two different aspects of this occupational risk were measured: exposure to the public per se and frequency of stressful exposure. Regarding stressful exposure, exploratory analyses provided some indications about jobs for which it could be particularly related to substances use. Although these results should be interpreted with caution due to limited sample sizes, greater risks in education and healthcare professionals are in line with the literature regarding a particularly important emotional demand among these workers [47, 48]. Finally, we were able to explore gender differences.

We observed greater odds ratios in men than in women when comparing exposed versus non-exposed workers. Firstly, these findings are in accordance with different reasons of substance use according to gender, especially regarding a greater tendency to use substances for socialization in men [49]. Secondly, when facing the public, men could be more prone to adjust their behaviors to conform to the social representations of masculinity, including some ease in substance use, or at least, not refusing to use them [49, 50]. Regarding the frequency of stressful exposure, we observed greater odds ratios in women than in men, which is in line with previous findings regarding the higher sensitivity of women to emotional job demand [17]. Moreover, gender differences may be explained by differences in the public encountered by men and women. First, women are more likely to occupy lower positions (i.e. low paid, precarious job), which tend to enhance both the intensity and the nature of the stressful relationships with the public, as the propensity to substance use disorders [2, 51]. Second, women more often occupy jobs that imply high emotional demand, such as caregiving or social working. Third, since men have higher rates of substance consumption than women, they will be more likely to be dismissed from stressful public exposure that could lead to a healthy worker effect disadvantaging women. Anyway, a better understanding of gender differences warrants further studies, especially to define at-risk subgroups in a public health perspective [29].”

Regarding the associations with smoking, since the entry to consumption most often preexist before entering in the labor market, these associations may be related to the fact that vulnerability to tobacco consumption and vulnerability to interpersonnal stress potentially share common pathways [52]. Indeed, the entry to tobacco consumption may be driven, at least for a subgroup of smokers, by attempts to reduce their social anxiety [52]. Regarding the association between stressful exposure and former smoking only in men, they might be explained, at least partially, by the potential increased vulnerability of former smokers to interpersonal stress. In former smokers, anxiety has been found to be more prevalent than in never smokers, including long after cessation [53]. Tobacco might also reduce perceived stress associated with job exposure in the public to some extent in men. However, further investigations are needed to explain why these associations with former smoking were seen solely in men.

### Clinical relevance and future research

Although relatively small effect sizes in the present results may challenge their relevance at an individual level, they may nonetheless inform preventive interventions which may have important public health implications given the high prevalence of both job positions with exposure to the public and addictive behaviors. Importantly, psychological interventions exist to reduce the emotional impact of exposure to the public [54, 55]. Our findings suggest that these interventions should systematically integrate the assessment and prevention measures of addictive behaviors. In addition, vulnerable individuals may be offered more specific interventions aiming at reducing the impact of interpersonal stressful relationships on their substance consumptions, with the advantage of addressing these issues into a non-stigmatizing stress management framework [56], or failing that, to reduce their exposure to the public. Given the prevalence and the huge health burden of addictive behaviors, such interventions are likely to result in substantial effects at a population level. Since job exposure to the public was associated with addictive behaviors regarding the three substances, targeting this risk factor could be of particular interest in preventing subsequent damages that are all the more severe that these conditions often co-occur.

Future researches are needed to evaluate the potential impact of preventive interventions targeting job exposure to the public on addictive behaviors and related burden. Since addictive behaviors may relate to different hazards (e.g. heavy episodic drinking is especially linked with external causes of death versus chronic consumption of alcohol or tobacco being linked with chronic diseases), future research should evaluate whether these preventive strategies may prove useful in reducing specific causes of death or disabilities. Our findings highlighted that job exposure to the public was associated not only with alcohol intake, but also with the relation to alcohol, in the way of a greater risk of alcohol use disorder. Since this aspect was not measured regarding tobaco and cannabis, further studies should examine tobacco and cannabis use disorders with the help of standardized assessments. Differences in magnitudes of the associations between job exposure to the public and substance use according to types of jobs should be further examined in order to target at-risk jobs and to refine prevention interventions taking into account job specificities, such as the type of public encountered. Gender differences also need to be better understood as well as subcomponents of stressful experiences related to exposure to the public (e.g. objective aspects of exposure such as frequency or intensity, subjective aspects of exposure such individual sensitivity to interpersonal conflicts) to help develop personalized psychological interventions.

## Supporting information

S1 Table Description of the responders to the assessments of job exposure to the public according to missing data regarding each dependent variable (n = 33,195). (DOCX)

S2 Table Exploratory age-adjusted analyses of the associations between substance use (alcohol, tobacco and cannabis) and frequency of stressful job exposure to the public in 7,865 men and 11,240 women, all daily exposed, and considering a frequent stressful exposure compared to a rare one while stratifying for types of job. (DOCX)

S3 Table Description of the covariables among the 33,992 included participants and according to imputations. (DOCX)

S1 Fig Flow chart. (DOCX)

## References

[1] WHO. Global Health Risks: Mortality and Burden of Disease Attributable to Selected Major Risks: World Health Organization; 2009.

[2] Redonnet B, Chollet A, Fombonne E, Bowes L, Melchior M. Tobacco, alcohol, cannabis and other illegal drug use among young adults: the socioeconomic context. Drug Alcohol Depend. 2012;121(3):231–9. doi: 10.1016/j.drugalcdep.2011.09.002 . 21955362 10.1016/j.drugalcdep.2011.09.002

[3] Corrao G, Bagnardi V, Zambon A, La Vecchia C. A meta-analysis of alcohol consumption and the risk of 15 diseases. Prev Med. 2004;38(5):613–9. Epub 2004/04/07. doi: 10.1016/j.ypmed.2003.11.027 . 15066364 10.1016/j.ypmed.2003.11.027

[4] Hall W, Degenhardt L. Adverse health effects of non-medical cannabis use. The Lancet. 2009;374(9698):1383–91. 10.1016/S0140-6736(09)61037-0 19837255

[5] Chen VC-H, Kuo C-J, Wang T-N, Lee W-C, Chen WJ, Ferri CP, et al Suicide and other-cause mortality after early exposure to smoking and second hand smoking: a 12-year population-based follow-up study. PloS one. 2015;10(7):e0130044 doi: 10.1371/journal.pone.0130044 26222448 10.1371/journal.pone.0130044 PMC4519334

[6] Blanco C, Hasin DS, Wall MM, Florez-Salamanca L, Hoertel N, Wang S, et al Cannabis Use and Risk of Psychiatric Disorders: Prospective Evidence From a US National Longitudinal Study. JAMA psychiatry. 2016;73(4):388–95. Epub 2016/02/18. doi: 10.1001/jamapsychiatry.2015.3229 . 26886046 10.1001/jamapsychiatry.2015.3229

[7] Wiesbeck GA, Kuhl HC, Yaldizli Ö, Wurst FM. Tobacco Smoking and Depression—Results from the WHO/ISBRA Study. Neuropsychobiology. 2008;57(1–2):26–31. doi: 10.1159/000123119 18424908 10.1159/000123119

[8] Sullivan LE, Fiellin DA, O’Connor PG. The prevalence and impact of alcohol problems in major depression: A systematic review. The American Journal of Medicine. 2005;118(4):330–41. https://doi.org/10.1016/j.amjmed.2005.01.007. 15808128 10.1016/j.amjmed.2005.01.007

[9] Shield KD, Rehm MX, Rehm J. Social costs of addiction in Europe In: Anderson P, Rehm J, and Room R (eds) The Impact of Addictive Substances and Behaviours on Individual and Societal Well-being. Oxford: Oxford University Press 2015:181–8.

[10] Morois S, Airagnes G, Lemogne C, Leclerc A, Limosin F, Goldberg S, et al Daily alcohol consumption and sickness absence in the GAZEL cohort. European journal of public health. 2017;27(3):482–8. doi: 10.1093/eurpub/ckx012 28339654 10.1093/eurpub/ckx012

[11] Weng SF, Ali S, Leonardi-Bee J. Smoking and absence from work: systematic review and meta-analysis of occupational studies. Addiction. 2013;108(2):307–19. Epub 2012/10/20. doi: 10.1111/add.12015 23078132 10.1111/add.12015

[12] Reed PL, Storr CL, Anthony JC. Drug Dependence Enviromics: Job Strain in the Work Environment and Risk of Becoming Drug-Dependent. American Journal of Epidemiology. 2006;163(5):404–11. doi: 10.1093/aje/kwj064 16421241 10.1093/aje/kwj064

[13] Lima CT, Farrell M, Prince M. Job strain, hazardous drinking, and alcohol-related disorders among Brazilian bank workers. Journal of studies on alcohol and drugs. 2013;74(2):212–22. 23384369 10.15288/jsad.2013.74.212

[14] Colell E, Sánchez‐Niubò A, Benavides FG, Delclos GL, Domingo-Salvany A. Work-related stress factors associated with problem drinking: A study of the Spanish working population. American journal of industrial medicine. 2014;57(7):837–46. doi: 10.1002/ajim.22333 24760618 10.1002/ajim.22333

[15] Nyberg ST, Fransson EI, Heikkilä K, Alfredsson L, Casini A, Clays E, et al Job strain and cardiovascular disease risk factors: meta-analysis of individual-participant data from 47,000 men and women. PloS one. 2013;8(6):e67323 doi: 10.1371/journal.pone.0067323 23840664 10.1371/journal.pone.0067323 PMC3688665

[16] Temple EC, Hammersley R, van Laar M, Brown RF. Clearing the smokescreen: The current evidence on cannabis use. Frontiers in Psychiatry. 2015;6(40). doi: 10.3389/fpsyt.2015.00040 25821437 10.3389/fpsyt.2015.00040 PMC4358058

[17] Rivera-Torres P, Araque-Padilla RA, Montero-Simó MJ. Job Stress across Gender: The Importance of Emotional and Intellectual Demands and Social Support in Women. International Journal of Environmental Research and Public Health. 2013;10(1):375–89. doi: 10.3390/ijerph10010375 23343989 10.3390/ijerph10010375 PMC3564148

[18] Head J, Stansfeld SA, Siegrist J. The psychosocial work environment and alcohol dependence: a prospective study. Occupational and Environmental Medicine. 2004;61(3):219–24. doi: 10.1136/oem.2002.005256 14985516 10.1136/oem.2002.005256 PMC1740737

[19] Sinha R. The role of stress in addiction relapse. Current Psychiatry Reports. 2007;9(5):388–95. doi: 10.1007/s11920-007-0050-6 17915078 10.1007/s11920-007-0050-6

[20] Frone MR. Work stress and alcohol use. Alcohol research and health. 1999;23(4):284–91. 10890825 PMC6760381

[21] Moore TM, Stuart GL. A review of the literature on marijuana and interpersonal violence. Aggression and Violent Behavior. 2005;10(2):171–92.

[22] WHO. Global status report on alcohol 2004: World Health Organization 2004.

[23] Kornreich C, Philippot P, Foisy ML, Blairy S, Raynaud E, Dan B, et al Impaired emotional facial expression recognition is associated with interpersonal problems in alcoholism. Alcohol and alcoholism (Oxford, Oxfordshire). 2002;37(4):394–400. Epub 2002/07/11. . 12107044 10.1093/alcalc/37.4.394

[24] Kidorf M, Lang AR. Effects of social anxiety and alcohol expectancies on stress-induced drinking. Psychology of Addictive Behaviors. 1999;13(2):134.

[25] Buckner JD, Eggleston AM, Schmidt NB. Social Anxiety and Problematic Alcohol Consumption: The Mediating Role of Drinking Motives and Situations. Behavior Therapy. 2006;37(4):381–91. https://doi.org/10.1016/j.beth.2006.02.007. 17071215 10.1016/j.beth.2006.02.007

[26] Buckner JD, Schmidt NB, Lang AR, Small JW, Schlauch RC, Lewinsohn PM. Specificity of social anxiety disorder as a risk factor for alcohol and cannabis dependence. Journal of Psychiatric Research. 2008;42(3):230–9. https://doi.org/10.1016/j.jpsychires.2007.01.002. 17320907 10.1016/j.jpsychires.2007.01.002 PMC2254175

[27] Ait-Daoud N, Blevins D, Khanna S, Sharma S, Holstege CP. Women and Addiction. Psychiatric Clinics of North America. 2017;40(2):285–97. https://doi.org/10.1016/j.psc.2017.01.005. 28477653 10.1016/j.psc.2017.01.005

[28] LaBrie JW, Hummer JF, Pedersen ER. Reasons for Drinking in the College Student Context: The Differential Role and Risk of the Social Motivator. Journal of studies on alcohol and drugs. 2007;68(3):393–8. doi: 10.15288/jsad.2007.68.393 17446979 10.15288/jsad.2007.68.393 PMC4214145

[29] Wang JL, Lesage A, Schmitz N, Drapeau A. The relationship between work stress and mental disorders in men and women: findings from a population-based study. Journal of epidemiology and community health. 2008;62(1):42–7. Epub 2007/12/15. doi: 10.1136/jech.2006.050591 . 18079332 10.1136/jech.2006.050591

[30] Hooftman WE, van der Beek AJ, Bongers PM, van Mechelen W. Gender Differences in Self-Reported Physical and Psychosocial Exposures in Jobs With Both Female and Male Workers. Journal of Occupational and Environmental Medicine. 2005;47(3):244–52. 15761320 10.1097/01.jom.0000150387.14885.6b

[31] Goldberg M, Carton M, Descatha A, Leclerc A, Roquelaure Y, Santin G, et al CONSTANCES: a general prospective population-based cohort for occupational and environmental epidemiology: cohort profile. Occup Environ Med. 2017;74(1):66–71. Epub 2016/11/26. doi: 10.1136/oemed-2016-103678 27884936 10.1136/oemed-2016-103678 PMC5241503

[32] Siegrist J, Li J. Associations of Extrinsic and Intrinsic Components of Work Stress with Health: A Systematic Review of Evidence on the Effort-Reward Imbalance Model. Int J Environ Res Public Health. 2016;13(4):432 Epub 2016/04/23. doi: 10.3390/ijerph13040432 27104548 10.3390/ijerph13040432 PMC4847094

[33] Gache P, Michaud P, Landry U, Accietto C, Arfaoui S, Wenger O, et al The Alcohol Use Disorders Identification Test (AUDIT) as a screening tool for excessive drinking in primary care: reliability and validity of a French version. Alcoholism, clinical and experimental research. 2005;29(11):2001–7. Epub 2005/12/13. . 16340457 10.1097/01.alc.0000187034.58955.64

[34] Babor TF, Higgins-Biddle JC, Saunders JB, Monteiro MG. AUDIT: The alcohol use disorders identification test: Guidelines for use in primary health care. 2 ed: World Health Organization; 2001.

[35] Lohse T, Rohrmann S, Bopp M, Faeh D. Heavy Smoking Is More Strongly Associated with General Unhealthy Lifestyle than Obesity and Underweight. PloS one. 2016;11(2):e0148563 doi: 10.1371/journal.pone.0148563 26910775 10.1371/journal.pone.0148563 PMC4765891

[36] Schneider SL. The International Standard Classification of Education 2011 In: Birkelund GE, editor. Class and Stratification Analysis (Comparative Social Research, Volume 30) Emerald Group Publishing Limited 2013 p. 365–79.

[37] Melchior M, Caspi A, Milne BJ, Danese A, Poulton R, Moffitt TE. Work stress precipitates depression and anxiety in young, working women and men. Psychological Medicine. 2007;37(8):1119–29. doi: 10.1017/S0033291707000414 17407618 10.1017/S0033291707000414 PMC2062493

[38] Fuhrer R, Rouillon F. La version française de l'échelle CES-D (Center for Epidemiologic Studies-Depression Scale). Description et traduction de l'échelle d’autoévaluation. European Psychiatry. 1989;4(3):163–66.

[39] Stewart SH, Connors GJ. Perceived health status, alcohol-related problems, and readiness to change among medically hospitalized, alcohol-dependent patients. Journal of Hospital Medicine. 2007;2(6):372–7. doi: 10.1002/jhm.211 18080338 10.1002/jhm.211

[40] Carton M S G, Geoffroy-Perez B, Chanut A. Contribution des variables annexes au codage des libellés de profession par le logiciel SICORE. Institut de veille sanitaire 2007:64.

[41] Winship C, Mare RD. Regression models with ordinal variables American sociological review. 1984:512–25.

[42] von Hippel PT. Regression with Missing Ys: An Improved Strategy for Analyzing Multiply Imputed Data. Sociological Methodology. 2007;37:83–117.

[43] Sabbath EL, Goldberg M, Wu Q, Descatha A. Can a single-item measure assess physical load at work? An analysis from the GAZEL cohort. Journal of Occupational and Environmental Medicine. 2012;54(5):598–603. 22481211 10.1097/JOM.0b013e31824af5a8 PMC3907304

[44] Haukoos JS, Newgard CD. Advanced Statistics: Missing Data in Clinical Research—Part 1: An Introduction and Conceptual Framework. Academic Emergency Medicine. 2007;14(7):662–8. 17538078 10.1197/j.aem.2006.11.037

[45] Grandey AA. Emotional regulation in the workplace: A new way to conceptualize emotional labor. Journal of occupational health psychology. 2000;5(1):95 10658889 10.1037//1076-8998.5.1.95

[46] Weissenborn R, Duka T. Acute alcohol effects on cognitive function in social drinkers: their relationship to drinking habits. Psychopharmacology. 2003;165(3):306–12. doi: 10.1007/s00213-002-1281-1 12439627 10.1007/s00213-002-1281-1

[47] Escribà-Agüir V, Martín-Baena D, Pérez-Hoyos S. Psychosocial work environment and burnout among emergency medical and nursing staff. International archives of occupational and environmental health. 2006;80(2):127–33. doi: 10.1007/s00420-006-0110-y 16710712 10.1007/s00420-006-0110-y

[48] Hakanen JJ, Bakker AB, Schaufeli WB. Burnout and work engagement among teachers. Journal of school psychology. 2006;43(6):495–513.

[49] Schulte MT, Ramo D, Brown SA. Gender differences in factors influencing alcohol use and drinking progression among adolescents. Clinical psychology review. 2009;29(6):535–47. https://doi.org/10.1016/j.cpr.2009.06.003. 19592147 10.1016/j.cpr.2009.06.003 PMC2756494

[50] McCreary DR, Newcomb MD, Sadava SW. The male role, alcohol use, and alcohol problems: A structural modeling examination in adult women and men. Journal of Counseling Psychology. 1999;46(1):109–24. doi: 10.1037/0022-0167.46.1.109

[51] Stier H, Yaish M. Occupational segregation and gender inequality in job quality: a multi-level approach. Work, employment and society. 2014;28(2):225–46. doi: 10.1177/0950017013510758

[52] Buckner JD, Vinci C. Smoking and Social Anxiety: The Roles of Gender and Smoking Motives. Addictive Behaviors. 2013;38(8):2388–91. doi: 10.1016/j.addbeh.2013.03.007 23639849 10.1016/j.addbeh.2013.03.007 PMC3677699

[53] Mykletun A, Overland S, Aarø LE, Liabø H-M, Stewart R. Smoking in relation to anxiety and depression: Evidence from a large population survey: The HUNT study. European Psychiatry. 23(2):77–84. doi: 10.1016/j.eurpsy.2007.10.005 18082377 10.1016/j.eurpsy.2007.10.005

[54] Hofmann SG. Cognitive Factors that Maintain Social Anxiety Disorder: a Comprehensive Model and its Treatment Implications. Cognitive Behaviour Therapy. 2007;36(4):193–209. doi: 10.1080/16506070701421313 18049945 10.1080/16506070701421313 PMC2151931

[55] Goldin PR, Gross JJ. Effects of Mindfulness-Based Stress Reduction (MBSR) on Emotion Regulation in Social Anxiety Disorder. Emotion (Washington, DC). 2010;10(1):83–91. doi: 10.1037/a0018441 20141305 10.1037/a0018441 PMC4203918

[56] Billings DW, Cook RF, Hendrickson A, Dove DC. A Web-Based Approach to Managing Stress and Mood Disorders in the Workforce. Journal of occupational and environmental medicine / American College of Occupational and Environmental Medicine. 2008;50(8):960–8. doi: 10.1097/JOM.0b013e31816c435b 18695455 10.1097/JOM.0b013e31816c435b PMC4570566

