# Supplementary material for: Job exposure to the public in relation with alcohol, tobacco and cannabis use: Findings from the CONSTANCES cohort study
Source: PLoS One. 2018 May 1;13(5):e0196330. doi: 10.1371/journal.pone.0196330 (PMC5929509; doi:10.1371/journal.pone.0196330)
Supplement: S1 Table — (DOCX) [file pone.0196330.s001.docx]

**S1 Table. Description of the responders to the assessments of job exposure to the public according to missing data regarding each dependent variable (n=33,195).**

|  | **Dependent variables** | | | | | | | | | | | | | | | | | | | | **Included** | | **Not included** | |
| --- | --- | --- | --- | --- | --- | --- | --- | --- | --- | --- | --- | --- | --- | --- | --- | --- | --- | --- | --- | --- | --- | --- | --- | --- |
|  | **Chronic alcohol consumption** | | | | **Heavy episodic drinking** | | | | **Alcohol use disorder risk level** | | | | **Tobacco consumption** | | | | **Cannabis consumption** | | | |  |  |  |  |
| **Sub-sample following data availability** | **Data available** | | **Missing data** | | **Data available** | | **Missing data** | | **Data available** | | **Missing data** | | **Data available** | | **Missing data** | | **Data available** | | **Missing data** | |  |  |  |  |
| N (%) | 42064(90.2) | | 4588(9.8) | | 40884(87.6) | | 5768(12.4) | | 38045(81.6) | | 8607(18.4) | | 43463(93.2) | | 3189(6.8) | | 43302(92.8) | | 3350(7.2) | | 33992(72.9) | | 12660(27.1) | |
|  |  | |  | |  | |  | |  | |  | |  | |  | |  |  |  |  |  |  |  |  |
| **CONTINUOUS COVARIABLES** | **Mean** | **SD** | **Mean** | **SD** | **Mean** | **SD** | **Mean** | **SD** | **Mean** | **SD** | **Mean** | **SD** | **Mean** | **SD** | **Mean** | **SD** | **Mean** | **SD** | **Mean** | **SD** | **Mean** | **SD** | **Mean** | **SD** |
| Age (years) | 43.6 | 10.3 | 43.8 | 10.4 | 43.7 | 10.3 | 43.0 | 10.5 | 43.5 | 10.3 | 44.1 | 10.4 | 43.6 | 10.3 | 44.9 | 10.1 | 43.5 | 10.3 | 47.0 | 10.6 | 43.5 | 10.2 | 44.0 | 10.5 |
| Perceived health status**^1^** | 2.7 | 1.3 | 2.9 | 1.5 | 2.7 | 1.3 | 2.9 | 1.5 | 2.7 | 1.3 | 2.9 | 1.4 | 2.7 | 1.3 | 2.7 | 1.3 | 2.7 | 1.3 | 2.8 | 1.4 | 2.7 | 1.3 | 2.8 | 1.4 |
| Effort-reward imbalance**^2^** | 1.1 | 0.4 | 1.1 | 0.5 | 1.1 | 0.4 | 1.1 | 0.5 | 1.1 | 0.4 | 1.1 | 0.5 | 1.1 | 0.4 | 1.1 | 0.5 | 1.1 | 0.4 | 1.1 | 0.5 | 1.1 | 0.4 | 1.1 | 0.5 |
| Depression score (CESD) | 10.4 | 8.4 | 12.2 | 9.5 | 10.3 | 8.3 | 12.3 | 9.5 | 10.2 | 8.2 | 12.1 | 9.4 | 10.4 | 8.4 | 11.3 | 9.0 | 10.4 | 8.4 | 11.7 | 8.7 | 10.1 | 8.2 | 11.6 | 9.1 |
|  |  |  |  |  |  |  |  |  |  |  |  |  |  |  |  |  |  |  |  |  |  |  |  |  |
| **CATEGORICAL COVARIABLES** | **N** | **%** | **N** | **%** | **N** | **%** | **N** | **%** | **N** | **%** | **N** | **%** | **N** | **%** | **N** | **%** | **N** | **%** | **N** | **%** | **N** | **%** | **N** | **%** |
| **Job exposure to the public** |  |  |  |  |  |  |  |  |  |  |  |  |  |  |  |  |  |  |  |  |  |  |  |  |
| Daily exposure | 29327 | 69.7 | 3140 | 68.4 | 28440 | 69.6 | 4027 | 69.8 | 26439 | 69.5 | 6028 | 70.0 | 30307 | 69.7 | 2160 | 67.7 | 30231 | 69.8 | 2236 | 66.7 | 23641 | 69.5 | 8826 | 69.7 |
| No exposure | 12737 | 30.3 | 1448 | 31.6 | 12444 | 30.4 | 1741 | 30.2 | 11606 | 30.5 | 2579 | 30.0 | 13156 | 30.3 | 1029 | 32.3 | 13071 | 30.2 | 1114 | 33.3 | 10351 | 30.5 | 3834 | 27.0 |
| **Gender** |  |  |  |  |  |  |  |  |  |  |  |  |  |  |  |  |  |  |  |  |  |  |  |  |
| Men | 19863 | 47.2 | 2034 | 44.3 | 19594 | 47.9 | 2303 | 39.9 | 18466 | 48.5 | 3431 | 39.9 | 20336 | 46.8 | 1561 | 48.9 | 20374 | 47.1 | 1523 | 45.5 | 16566 | 48.7 | 5331 | 42.1 |
| Women | 22201 | 52.8 | 2554 | 55.7 | 21290 | 52.1 | 3465 | 60.1 | 19579 | 51.5 | 5176 | 60.1 | 23127 | 53.2 | 1628 | 51.1 | 22928 | 52.9 | 1827 | 54.5 | 17426 | 51.3 | 7329 | 57.9 |
| **Occupational status^3^** |  |  |  |  |  |  |  |  |  |  |  |  |  |  |  |  |  |  |  |  |  |  |  |  |
| Farmer, blue-collar worker and craftsman | 2108 | 7.2 | 341 | 10.9 | 2041 | 7.2 | 408 | 10.1 | 1829 | 6.9 | 620 | 10.3 | 2259 | 7.5 | 190 | 8.8 | 2231 | 7.4 | 218 | 9.7 | 1602 | 6.8 | 847 | 9.6 |
| Clerk | 8703 | 29.7 | 1221 | 38.9 | 8285 | 29.1 | 1639 | 40.7 | 7479 | 28.3 | 2445 | 40.6 | 9269 | 30.6 | 655 | 30.3 | 9130 | 30.2 | 794 | 35.5 | 6670 | 28.2 | 3254 | 36.9 |
| Intermediate worker | 10092 | 34.4 | 943 | 30.0 | 9814 | 34.5 | 1221 | 30.3 | 9230 | 34.9 | 1805 | 29.9 | 10327 | 34.1 | 708 | 32.8 | 10337 | 34.2 | 698 | 31.2 | 8336 | 35.3 | 2699 | 30.6 |
| Executive | 8424 | 28.7 | 635 | 20.2 | 8300 | 29.2 | 759 | 18.8 | 7901 | 29.9 | 1158 | 19.2 | 8452 | 27.9 | 607 | 28.1 | 8533 | 28.2 | 526 | 23.5 | 7033 | 29.7 | 2026 | 23.0 |
| **Marital status^3^** |  |  |  |  |  |  |  |  |  |  |  |  |  |  |  |  |  |  |  |  |  |  |  |  |
| Single | 6324 | 15.2 | 500 | 16.6 | 6085 | 15.1 | 739 | 17.6 | 5663 | 15.1 | 1161 | 16.7 | 6569 | 15.3 | 255 | 15.0 | 6519 | 15.3 | 305 | 16.5 | 4980 | 14.8 | 1844 | 16.9 |
| Marital life | 30372 | 73.2 | 2072 | 68.6 | 29559 | 73.3 | 2885 | 68.7 | 27635 | 73.5 | 4809 | 69.2 | 31246 | 72.9 | 1198 | 70.3 | 31185 | 73.1 | 1259 | 68.0 | 24845 | 73.9 | 7599 | 69.5 |
| Separated or divorced | 4396 | 10.6 | 396 | 13.1 | 4277 | 10.6 | 515 | 12.3 | 3915 | 10.4 | 877 | 12.6 | 4558 | 10.6 | 234 | 13.7 | 4541 | 10.6 | 251 | 13.6 | 3449 | 10.3 | 1343 | 12.3 |
| Widower | 426 | 1.0 | 53 | 1.8 | 421 | 1.0 | 58 | 1.4 | 379 | 1.0 | 100 | 1.4 | 461 | 1.1 | 18 | 1.1 | 443 | 1.0 | 36 | 1.9 | 331 | 1.0 | 148 | 1.4 |
| **Household income** (euros per month) **^3^** |  |  |  |  |  |  |  |  |  |  |  |  |  |  |  |  |  |  |  |  |  |  |  |  |
| Less than 2100 | 6918 | 17.3 | 933 | 33.2 | 6587 | 17.0 | 1264 | 31.8 | 5911 | 16.3 | 1940 | 29.9 | 7519 | 18.3 | 332 | 20.5 | 7360 | 17.9 | 491 | 29.3 | 5155 | 15.9 | 2696 | 26.1 |
| Between 2100 and 2800 | 6377 | 16.0 | 524 | 18.6 | 6107 | 15.7 | 794 | 20.0 | 5615 | 15.5 | 1286 | 19.8 | 6646 | 16.1 | 255 | 15.7 | 6588 | 16.0 | 313 | 18.7 | 5009 | 15.4 | 1892 | 18.4 |
| Between 2800 and 4200 | 13446 | 33.6 | 797 | 28.4 | 13082 | 33.7 | 1161 | 29.3 | 12331 | 34.0 | 1912 | 29.4 | 13743 | 33.4 | 500 | 30.8 | 13774 | 33.5 | 469 | 28.0 | 11114 | 34.2 | 3129 | 30.3 |
| More than 4200 | 13235 | 33.1 | 557 | 19.8 | 13042 | 33.6 | 750 | 18.9 | 12433 | 34.3 | 1359 | 20.9 | 13258 | 32.2 | 534 | 32.9 | 13391 | 32.6 | 401 | 24.0 | 11199 | 34.5 | 2593 | 25.2 |
| **Education ISCED classification ^3^** |  |  |  |  |  |  |  |  |  |  |  |  |  |  |  |  |  |  |  |  |  |  |  |  |
| Level 0 and level 1 | 804 | 1.9 | 202 | 6.6 | 757 | 1.9 | 249 | 5.9 | 600 | 1.6 | 406 | 5.8 | 959 | 2.2 | 47 | 2.7 | 909 | 2.1 | 97 | 5.2 | 202 | 6.6 | 804 | 1.9 |
| Level 2 | 1527 | 3.7 | 217 | 7.1 | 1479 | 3.6 | 265 | 6.3 | 1272 | 3.4 | 472 | 6.7 | 1669 | 3.9 | 75 | 4.4 | 1602 | 3.7 | 142 | 7.6 | 217 | 7.1 | 1527 | 3.7 |
| Level 3 and level 4 | 12538 | 30.1 | 1245 | 40.9 | 12115 | 29.9 | 1668 | 39.5 | 10946 | 29.0 | 2837 | 40.5 | 13218 | 30.7 | 565 | 33.0 | 13044 | 30.4 | 739 | 39.8 | 1245 | 40.9 | 12538 | 30.1 |
| Level 5 and level 6 | 15628 | 37.5 | 895 | 29.4 | 15180 | 37.4 | 1343 | 31.8 | 14370 | 38.1 | 2153 | 30.8 | 15914 | 37.0 | 609 | 35.6 | 15993 | 37.3 | 530 | 28.5 | 895 | 29.4 | 15628 | 37.5 |
| Level 7 and level 8 | 11222 | 26.9 | 483 | 15.9 | 11012 | 27.2 | 693 | 16.4 | 10572 | 28.0 | 1133 | 16.2 | 11288 | 26.2 | 417 | 24.3 | 11354 | 26.5 | 351 | 18.9 | 483 | 15.9 | 11222 | 26.9 |

SD: Standard Deviation; N: Number of subjects; CESD: Center for Epidemiologic Studies Depression Scale; ISCED: International Standard Classification of Education; **^1^**From a 8-points Likert scale with a score of 1 indicated a very good general health and a score of 8 a very poor one; ^2^Computed from 7 items regarding rewards and from 3 items regarding efforts as follows: ERI= (7/3)*(effort total score/reward total score), and with all the items assessed on a 4-points likert scale; ^3^Parameters of the covariables in sub-samples without missing data for the given covariable.
