# Supplementary material for: Job exposure to the public in relation with alcohol, tobacco and cannabis use: Findings from the CONSTANCES cohort study
Source: PLoS One. 2018 May 1;13(5):e0196330. doi: 10.1371/journal.pone.0196330 (PMC5929509; doi:10.1371/journal.pone.0196330)
Supplement: S2 Table — (DOCX) [file pone.0196330.s002.docx]

**S2 Table. Exploratory age-adjusted analyses of the associations between substance use (alcohol, tobacco and cannabis) and frequency of stressful job exposure to the public in 7,865 men and 11,240 women, all daily exposed, and considering a frequent stressful exposure compared to a rare one while stratifying for types of job.**

| **TYPE OF JOBS** | **Administrative staff** | | | **Education professions** | | | **Healthcare professions** | | | **Supervisors, workers and technicians not in tertiary** | | | **Engineers** | | | **Personal services** | | | **Commercial and independent professions** | | |
| --- | --- | --- | --- | --- | --- | --- | --- | --- | --- | --- | --- | --- | --- | --- | --- | --- | --- | --- | --- | --- | --- |
| **N** | **753** | | | **1357** | | | **554** | | | **1609** | | | **660** | | | **741** | | | **1908** | | |
| **MEN** | **OR** | **95%CI** | | **OR** | **95%CI** | | **OR** | **95%CI** | | **OR** | **95%CI** | | **OR** | **95%CI** | | **OR** | **95%CI** | | **OR** | **95%CI** | |
|  |  |  |  |  |  |  |  |  |  |  |  |  |  |  |  |  |  |  |  |  |  |
| **Chronic alcohol consumption^a^** |  |  |  |  |  |  |  |  |  |  |  |  |  |  |  |  |  |  |  |  |  |
| Light | 1.24 | 0.82 | 1.87 | 1.13 | 0.86 | 1.48 | 0.73 | 0.49 | 1.08 | 1.03 | 0.78 | 1.38 | 1.26 | 0.82 | 1.94 | **0.66 (p=0.014)** | **0.47** | **0.92** | 0.95 | 0.74 | 1.20 |
| Moderate | 1.07 | 0.38 | 3.06 | 1.37 | 0.73 | 2.57 | 0.37 | 0.10 | 1.32 | 0.65 | 0.32 | 1.31 | 1.92 | 0.76 | 4.86 | 1.13 | 0.58 | 2.20 | 0.72 | 0.43 | 1.20 |
| High or very high | 0.44 | 0.05 | 3.52 | 2.21 | 0.93 | 5.28 | 2.68 | 0.43 | 16.83 | 1.19 | 0.60 | 2.36 | 1.33 | 0.39 | 4.54 | 0.15 | 0.02 | 1.15 | 0.69 | 0.29 | 1.66 |
|  |  |  |  |  |  |  |  |  |  |  |  |  |  |  |  |  |  |  |  |  |  |
| **Heavy episodic drinking^b^** |  |  |  |  |  |  |  |  |  |  |  |  |  |  |  |  |  |  |  |  |  |
| At most once a month | **1.72 (p=0.006)** | **1.17** | **2.53** | 1.18 | 0.91 | 1.52 | 0.99 | 0.67 | 1.46 | 0.88 | 0.67 | 1.16 | 1.05 | 0.70 | 1.56 | **0.71 (p=0.05)** | **0.50** | **1.00** | 0.99 | 0.79 | 1.25 |
| More than once a month | 1.45 | 0.68 | 3.12 | **2.90 (p<0.001)** | **1.81** | **4.65** | 1.03 | 0.51 | 2.09 | 1.31 | 0.87 | 1.98 | 1.31 | 0.70 | 2.43 | 1.08 | 0.62 | 1.87 | 0.88 | 0.62 | 1.24 |
|  |  |  |  |  |  |  |  |  |  |  |  |  |  |  |  |  |  |  |  |  |  |
| **Alcohol use risk^c^** |  |  |  |  |  |  |  |  |  |  |  |  |  |  |  |  |  |  |  |  |  |
| Dangerous | 1.01 | 0.60 | 1.70 | **1.48 (p=0.009)** | **1.10** | **2.00** | 1.44 | 0.90 | 2.29 | 0.80 | 0.58 | 1.11 | 0.95 | 0.61 | 1.47 | 0.87 | 0.58 | 1.31 | 0.99 | 0.78 | 1.28 |
| Problematic or Dependence | 1.29 | 0.41 | 4.05 | **3.81 (p=0.001)** | **1.79** | **8.09** | 1.43 | 0.51 | 4.01 | 0.79 | 0.37 | 1.72 | 1.07 | 0.37 | 3.12 | 1.66 | 0.70 | 3.93 | 0.81 | 0.48 | 1.37 |
|  |  |  |  |  |  |  |  |  |  |  |  |  |  |  |  |  |  |  |  |  |  |
| **Smoking status^d^** |  |  |  |  |  |  |  |  |  |  |  |  |  |  |  |  |  |  |  |  |  |
| Former smoker | **1.55 (p=0.031)** | **1.04** | **2.31** | **1.42 (p=0.009)** | **1.09** | **1.84** | 0.96 | 0.64 | 1.45 | 1.09 | 0.82 | 1.44 | 1.35 | 0.93 | 1.98 | 1.07 | 0.85 | 1.34 | 1.07 | 085 | 1.34 |
| Light smoker | 0.89 | 0.38 | 2.08 | 1.53 | 0.97 | 2.42 | 0.87 | 0.43 | 1.76 | 0.91 | 0.55 | 1.51 | 0.42 | 0.16 | 1.10 | 0.78 | 0.53 | 1.15 | 0.78 | 0.53 | 1.15 |
| Moderate smoker | 1.67 | 0.82 | 3.40 | 1.30 | 0.75 | 2.23 | 1.34 | 0.71 | 2.51 | 0.77 | 0.49 | 1.20 | **0.36 (p=0.041)** | **0.14** | **0.96** | 0.93 | 0.65 | 1.34 | 0.93 | 0.65 | 1.34 |
| Heavy smoker | 2.43 | 0.80 | 7.40 | 1.51 | 0.69 | 3.31 | 0.60 | 0.12 | 3.00 | 0.71 | 0.38 | 1.33 | 1.05 | 0.39 | 2.83 | 1.07 | 0.63 | 1.81 | 1.07 | 0.63 | 1.81 |
|  |  |  |  |  |  |  |  |  |  |  |  |  |  |  |  |  |  |  |  |  |  |
| **Cannabis consumption^e^** |  |  |  |  |  |  |  |  |  |  |  |  |  |  |  |  |  |  |  |  |  |
| Consumption more than 12 months ago | 1.33 | 0.91 | 1.93 | **1.31 (p=0.035)** | **1.02** | **1.69** | 1.22 | 0.82 | 1.83 | 1.04 | 0.80 | 1.36 | 1.17 | 0.80 | 1.70 | 1.11 | 0.80 | 1.55 | 1.20 | 0.97 | 1.49 |
| Less than once a month | 0.88 | 0.29 | 2.66 | 1.39 | 0.80 | 2.40 | 1.60 | 0.78 | 3.31 | 0.75 | 0.38 | 1.48 | 1.01 | 0.48 | 2.13 | 0.87 | 0.35 | 2.14 | 1.06 | 0.67 | 1.66 |
| Once a month or more | 0.62 | 0.18 | 2.16 | **2.15 (p=0.003)** | **1.30** | **3.57** | 1.06 | 0.50 | 2.25 | 1.11 | 0.65 | 1.90 | 0.64 | 0.28 | 1.46 | 0.67 | 0.34 | 1.29 | 0.82 | 0.53 | 1.27 |
|  |  |  |  |  |  |  |  |  |  |  |  |  |  |  |  |  |  |  |  |  |  |
| **N** | **1659** | | | **2367** | | | **2486** | | | **371** | | | **211** | | | **1437** | | | **2226** | | |
| **WOMEN** | **OR** | **95%CI** | | **OR** | **95%CI** | | **OR** | **95%CI** | | **OR** | **95%CI** | | **OR** | **95%CI** | | **OR** | **95%CI** | | **OR** | **95%CI** | |
|  |  |  |  |  |  |  |  |  |  |  |  |  |  |  |  |  |  |  |  |  |  |
| **Chronic alcohol consumption^a^** |  |  |  |  |  |  |  |  |  |  |  |  |  |  |  |  |  |  |  |  |  |
| Light | 1.02 | 0.81 | 1.29 | 0.93 | 0.78 | 1.11 | 1.00 | 0.85 | 1.18 | 1.36 | 0.80 | 2.32 | 0.83 | 0.44 | 1.54 | 1.17 | 0.92 | 1.49 | 0.90 | 0.74 | 1.09 |
| Moderate | 1.55 | 0.90 | 2.68 | 1.06 | 0.75 | 1.52 | 1.42 | 0.97 | 2.09 | 1.71 | 0.57 | 5.09 | 1.29 | 0.42 | 3.96 | 0.94 | 0.53 | 1.67 | 0.84 | 0.57 | 1.22 |
| High or very high | 1.21 | 0.32 | 4.61 | **2.69 (p=0.040)** | **1.05** | **6.91** | 0.88 | 0.35 | 2.19 | 1.21 | 0.13 | 11.23 | . | . | . | 2.13 | 0.67 | 6.79 | **2.15 (p=0.031)** | **1.07** | **4.32** |
|  |  |  |  |  |  |  |  |  |  |  |  |  |  |  |  |  |  |  |  |  |  |
| **Heavy episodic drinking^b^** |  |  |  |  |  |  |  |  |  |  |  |  |  |  |  |  |  |  |  |  |  |
| At most once a month | 1.18 | 0.92 | 1.53 | 1.06 | 0.88 | 1.29 | 1.07 | 0.89 | 1.29 | 0.59 | 0.33 | 1.05 | 1.46 | 0.78 | 2.75 | 1.10 | 0.84 | 1.43 | 0.99 | 0.82 | 1.22 |
| More than once a month | 1.02 | 0.49 | 2.10 | 1.50 | 0.85 | 2.63 | 1.17 | 0.89 | 2.32 | 3.47 | 0.83 | 14.53 | 0.86 | 0.16 | 4.55 | 1.34 | 0.70 | 2.58 | 1.30 | 0.81 | 2.07 |
|  |  |  |  |  |  |  |  |  |  |  |  |  |  |  |  |  |  |  |  |  |  |
| **Alcohol use risk^c^** |  |  |  |  |  |  |  |  |  |  |  |  |  |  |  |  |  |  |  |  |  |
| Dangerous | 1.25 | 0.76 | 2.06 | **1.83 (p=0.001)** | **1.28** | **2.63** | **1.61 (p=0.006)** | **1.15** | **2.27** | 1.81 | 0.84 | 3.86 | 0.80 | 0.24 | 2.62 | 0.87 | 0.50 | 1.53 | 1.14 | 0.82 | 1.58 |
| Problematic or Dependence | 2.04 | 0.34 | 12.32 | **5.21 (p=0.014)** | **1.40** | **19.32** | 1.73 | 0.50 | 6.00 | . | . | . | . | . | . | 2.77 | 0.80 | 9.65 | 1.49 | 0.70 | 3.17 |
|  |  |  |  |  |  |  |  |  |  |  |  |  |  |  |  |  |  |  |  |  |  |
| **Smoking status^d^** |  |  |  |  |  |  |  |  |  |  |  |  |  |  |  |  |  |  |  |  |  |
| Former smoker | 1.19 | 0.93 | 1.54 | 1.07 | 0.89 | 1.29 | 1.09 | 0.91 | 1.32 | 1.72 | 0.93 | 3.18 | 1.22 | 0.61 | 2.44 | 1.07 | 0.81 | 1.40 | 1.01 | 0.82 | 1.25 |
| Light smoker | 0.97 | 0.63 | 1.49 | 1.05 | 0.75 | 1.46 | 1.26 | 0.95 | 1.68 | 1.32 | 0.56 | 3.08 | 1.08 | 0.38 | 3.05 | 0.96 | 0.64 | 1.45 | 1.07 | 0.79 | 1.45 |
| Moderate smoker | 1.12 | 0.74 | 1.70 | 1.33 | 0.89 | 1.99 | **1.77 (p<0.001)** | **1.30** | **2.42** | 2.27 | 0.99 | 5.17 | 2.11 | 0.60 | 7.43 | 0.94 | 0.62 | 1.42 | 1.03 | 0.75 | 1.42 |
| Heavy smoker | **2.27 (p=0.05)** | **1.00** | **5.14** | 1.56 | 0.68 | 3.58 | **2.51 (p=0.005)** | **1.32** | **4.75** | 1.96 | 0.49 | 7.81 | 3.39 | 0.53 | 21.83 | 1.04 | 0.49 | 2.19 | 1.76 | 0.99 | 3.12 |
|  |  |  |  |  |  |  |  |  |  |  |  |  |  |  |  |  |  |  |  |  |  |
| **Cannabis consumption^e^** |  |  |  |  |  |  |  |  |  |  |  |  |  |  |  |  |  |  |  |  |  |
| Consumption more than 12 months ago | 1.11 | 0.87 | 1.42 | **1.23 (p=0.025)** | **1.03** | **1.46** | **1.48 (p<0.001)** | **1.25** | **1.76** | 1.13 | 0.66 | 1.93 | 1.56 | 0.83 | 2.95 | 1.07 | 0.83 | 1.37 | 1.13 | 0.93 | 1.38 |
| Less than once a month | 1.08 | 0.51 | 2.28 | 0.90 | 0.53 | 1.51 | 1.15 | 0.72 | 1.86 | . | . | . | 0.94 | 0.24 | 3.77 | 1.64 | 0.85 | 3.16 | 1.01 | 0.63 | 1.63 |
| Once a month or more | 1.21 | 0.42 | 3.44 | 1.35 | 0.71 | 2.58 | **2.02 (p=0.010)** | **1.19** | **3.44** | 1.95 | 0.63 | 6.05 | . | . | . | 0.66 | 0.28 | 1.55 | 164 | 0.68 | 2.74 |

OR: Odd ratio; 95%CI: Confidence interval at 95%; ^a^Reference category is non-regular consumption, i.e. consumption of less than one standard drink per week. Regarding regular consumers, the following cut-offs in men(women):<28drinks per week(14); <43(29); <71(43) and ≥71(43) define low, medium, high or very high risk alcohol consumption categories, respectively; ^b^ Defined as at least six standard alcoholic beverages on the same occasion and taking the "never" category as reference; ^c^ Categories are defined from Alcohol Use Disorders Identification scores as follows: Mild (0-7), Dangerous (8-15), Problematic (16-19) and Dependence (20-40), with Mild category as reference; ^d^ Categories of current smokers are defined as follows: Light (1 to 9 cigarettes per day), Moderate (10 to 19) and Heavy (>19) consumers, with non-smokers as reference category; ^e^ Reference category is never use. Age at baseline was used as continuous covariable. Significant associations are presented in bold (i.e. p<0.05).
