# Supplementary material for: Job exposure to the public in relation with alcohol, tobacco and cannabis use: Findings from the CONSTANCES cohort study
Source: PLoS One. 2018 May 1;13(5):e0196330. doi: 10.1371/journal.pone.0196330 (PMC5929509; doi:10.1371/journal.pone.0196330)
Supplement: S3 Table — (DOCX) [file pone.0196330.s003.docx]

**S3 Table. Description of the covariables among the 33,992 included participants and according to imputations.**

| **GENDER** | **MEN** | | | | **WOMEN** | | | |
| --- | --- | --- | --- | --- | --- | --- | --- | --- |
| **N (%)** | **16,566(48.7%)** | | | | **17,426(51.3%)** | | | |
|  | **Included^a^** | | **Before imputation^b^** | | **Included^a^** | | **Before imputation^b^** | |
| **CONTINUOUS VARIABLES** | **Mean** | **SD** | **Mean** | **SD** | **Mean** | **SD** | **Mean** | **SD** |
| Age (years) | 44.3 | 10.3 | 44.3 | 10.3 | 43.7 | 10.4 | 43.7 | 10.4 |
| Depression score (CESD) | 9.0 | 7.3 | 8.9 | 7.3 | 11.4 | 8.9 | 11.4 | 8.9 |
| Perceived health status^c^ | 2.7 | 1.3 | 2.7 | 1.3 | 2.7 | 1.3 | 2.7 | 1.3 |
| Effort-reward imbalance^d^ | 1.0 | 0.4 | 1.0 | 0.4 | 1.1 | 0.4 | 1.1 | 0.4 |
| **CATEGORICAL VARIABLES** | **N** | **%** | **N** | **%** | **N** | **%** | **N** | **%** |
| **Occupational status** |  |  |  |  |  |  |  |  |
| Farmer, blue-collar worker and craftsman | 2512 | 15.2 | 2512 | 15.2 | 558 | 3.2 | 558 | 3.2 |
| Clerk | 2382 | 14.4 | 2382 | 14.4 | 5801 | 33.3 | 5801 | 33.3 |
| Intermediate worker | 4358 | 26.3 | 4358 | 26.3 | 5948 | 34.1 | 5948 | 34.1 |
| Executive | 7314 | 44.2 | 7314 | 44.2 | 5119 | 29.4 | 5119 | 29.4 |
| **Marital status** |  |  |  |  |  |  |  |  |
| Single | 2341 | 14.1 | 2311 | 14.1 | 2707 | 15.5 | 2669 | 15.5 |
| Marital life | 12768 | 77.1 | 12616 | 77.1 | 12343 | 70.8 | 12229 | 70.9 |
| Separated or divorced | 1380 | 8.3 | 1363 | 8.3 | 2112 | 12.1 | 2086 | 12.1 |
| Widower | 77 | 0.5 | 77 | 0.5 | 264 | 1.5 | 254 | 1.5 |
| **Household income** (euros per month) |  |  |  |  |  |  |  |  |
| Less than 2100 | 2252 | 13.6 | 2133 | 13.4 | 3191 | 18.3 | 3022 | 18.2 |
| Between 2100 and 2800 | 2407 | 14.5 | 2304 | 14.5 | 2854 | 16.4 | 2705 | 16.3 |
| Between 2800 and 4200 | 5708 | 34.5 | 5474 | 34.4 | 5392 | 34.0 | 5640 | 34.0 |
| More than 4200 | 6199 | 37.4 | 5993 | 37.7 | 5449 | 31.3 | 5206 | 31.4 |
| **Education ISCED classification** |  |  |  |  |  |  |  |  |
| Level 0 and level 1 | 302 | 1.8 | 302 | 1.8 | 217 | 1.2 | 217 | 1.3 |
| Level 2 | 551 | 3.3 | 539 | 3.3 | 583 | 3.3 | 572 | 3.3 |
| Level 3 and level 4 | 5232 | 31.6 | 5201 | 31.6 | 4559 | 26.2 | 4526 | 26.2 |
| Level 5 and level 6 | 5372 | 32.4 | 5320 | 32.3 | 7668 | 44.0 | 7614 | 44.0 |
| Level 7 and level 8 | 5109 | 30.8 | 5086 | 30.9 | 4399 | 25.2 | 4376 | 25.3 |

SD: Standard Deviation; CESD: Center for Epidemiologic Studies Depression Scale; ISCED: International Standard Classification of Education; ^a^ Characteristics of included participants after imputation for missing data in covariables; ^b^ Parameters of the covariables in sub-samples without missing data; ^c^ From a 8-points Likert scale with a score of 1 indicated a very good general health and a score of 8 a very poor one; ^d^ Computed from 7 items regarding rewards and from 3 items regarding efforts as follows: ERI= (7/3)*(effort total score/reward total score), and with all the items assessed on a 4-points likert scale.
