# Supplementary material for: Job exposure to the public in relation with alcohol, tobacco and cannabis use: Findings from the CONSTANCES cohort study
Source: PLoS One. 2018 May 1;13(5):e0196330. doi: 10.1371/journal.pone.0196330 (PMC5929509; doi:10.1371/journal.pone.0196330)
Supplement: S1 Fig — (DOCX) [file pone.0196330.s004.docx]

**S1 Fig. Flow chart**

**Participants with a daily job exposure to the public (n=23,641)**

**Participants without a daily job exposure to the public (n=10,351)**
